# National Hospital for the Paralysed and Epileptic

**Published:** 1904-12-17

**Authors:** Thomas Buzzard, W. R. Gowers, Victor Horsley, J. Danvers Power

**Affiliations:** President. Queen Square, Bloomsbury, W.C.; Treasurer. Queen Square, Bloomsbury, W.C.; Queen Square, Bloomsbury, W.C.; Queen Square, Bloomsbury, W.C.; Queen Square, Bloomsbury, W.C.; Chairman. Queen Square, Bloomsbury, W.C.


### Editor's Letter-Box.

[Our Correspondents are reminded that prolixity is a great bar to publication, and that brevity of style and conciseness of statement greatly facilitate early insertion.]

### NATIONAL HOSPITAL FOR THE PARALYSED AND EPILEPTIC.

*To the Editor of* The Hospital.

Sir,—The Hospital already does so much in the way of assisting deserving charities to make known their pressing needs, and this hospital has been so frequently before the public in consequence of its insufficient income, that we feel great reluctance in trespassing upon your space.

The maximum income we can depend upon is £13,000 a year. The very lowest expenditure we can estimate is £19,000 a year. The Board of Management now find themselves without anything upon which they can rely, as all capital available for general purposes has been—or before December 31st this year will have been—either expended or pledged.

We therefore begin next year with the knowledge that we must cut our expenses down by £6,000 a year, and as the first step, it has been decided to close the Convalescent Home at East Finchley, which does a most important part of our work, but which costs £2,000 a year. The closing of wards must inevitably follow unless the public come to our relief.

Should this letter be read by a rich man who desires to do a great work, he would find an opportunity in the taking over of our Convalescent Home and the avoidance of this reduction of the hospital's usefulness. Or should a sufficient number of people come forward to guarantee £2,000 a year for the purpose of keeping the Convalescent Home open, the same object would be equally well accomplished.

Failing this support some provision might be made for the patients elsewhere. The unfortunate people who suffer from epilepsy and paralysis are in many cases even more in need of a convalescent home than other classes of sufferers, and yet convalescent homes, as a rule, are not able to take them. Now that they are to be driven off the little anchorage they have at East Finchley we should indeed be glad if a few beds could be reserved for them under some other management.

Your obedient servants,

Dudley, President.
Harrowby, Treasurer.
Thomas Buzzard, M.D.
W. R. Gowers.
Victor Horsley.
J. Danvers Power, Chairman.

Queen Square, Bloomsbury, W.C.

